# Serological and molecular detection of *Toxoplasma Gondii* among cancer patients in Sohag, Upper Egypt: a case-control study

**DOI:** 10.1038/s41598-025-88680-3

**Published:** 2025-02-12

**Authors:** Eman Fathi Fadel, Mohammed Essa Marghany Tolba, Amal Mostafa Ahmed, Hanaa Ahmed EL-Hady

**Affiliations:** 1 Department of Medical Parasitology, Faculty of Medicine, Sohag University, Sohag, Egypt; 2 Department of Microbiology and Clinical Parasitology, Faculty of Medicine, King Khaled University, Abha, Saudi Arabia

**Keywords:** *Toxoplasma Gondii*, Serology, Molecular, Cancer patients, Egypt, Parasitology, Parasite genomics, Parasite host response

## Abstract

*Toxoplasma gondii (T. gondii)* affects around 30% of humans worldwide. Recently, it has emerged as a significant opportunistic pathogen to immunocompromised patients. Data available is still lacking about toxoplasmosis in cancer patients in Egypt. This study aimed to reveal the current trend of *T. gondii* in cancer patients in Sohag, Egypt. Sera from 50 cancer patients and 50 healthy controls were screened for *Toxoplasma* IgG and IgM. Further, buffy coats from both groups were used for detection of *T. gondii* B1 and RE genes via conventional and nested PCR, respectively. The overall seroprevalence of *T. gondii* IgG was high (58%). IgG and IgM were detected in 30% and 9% cancer patients, respectively. Patients with solid cancers exhibited a greater IgG seropositivity compared to those with hematologic tumors (77.27% and 46.43%, respectively) (*P* = 0.03). Concerning the molecular results, only 4 (9%) were positive regarding both PCR assays. In conclusion, *T. gondii* is highly prevalent in cancer patients in Sohag, Egypt. PCR is strongly recommended to complement serology to diagnose acute or reactivated toxoplasmosis in cancer patients. B1 PCR was found to be equivalent to RE PCR. Nevertheless, thorough large-scale research must be implemented.

**Supplementary Information:**

The online version contains supplementary material available at 10.1038/s41598-025-88680-3.

## Introduction

*Toxoplasma gondii* (*T. gondii*) is an intracellular protozoan parasite that can infect any nucleated vertebrate cells. It affects 30–65% of the human population worldwide^1,2^. Humans become primarily infected by ingesting raw or undercooked meat containing viable tissue cysts, or by ingesting water or food contaminated with oocysts from infected cat feces^3^. The primary infection in immunocompetent individuals is typically either asymptomatic or oligosymptomatic with lymphadenopathy and flu-like symptoms, depending on host susceptibility and the virulence of the strain^4,5^. *T. gondii* has recently emerged as a significant opportunistic pathogen that poses a life-threatening risk to immunocompromised patients. Cancer patients are exposed to a risk of reactivating a latent infection as a result of deficient cellular immunity or antineoplastic drugs. Cancer chemotherapy induces profound and persistent CD4 T cells depletion^6^. *T. gondii* disseminates through the host body with using the Trojan horse strategy, it can even enter immune-privileged sites resulting in toxoplasmic chorioretinitis and encephalitis following reactivation^7^. Thus, effective, rapid and accurate diagnosis is crucial and desirable to initiate adequate treatment and to achieve good prognosis^8^. The diagnosis of *T. gondii* infection continues to be a major challenge. Currently, serological diagnosis plays a crucial role in the diagnosis of *T. gondii* human infections^9^. Despite the innovations in serologic methods, there are still intractable limitations including the inability to confirm the existence of the parasite in immunocompromised patients^10^. To tackle these constraints, a variety of molecular assays such as conventional, nested, real-time PCR (qPCR) and loop-mediated isothermal amplification (LAMP) have been developed to detect *T. gondii* DNA in different biological samples^11^. Since molecular diagnosis is not influenced by the immunologic status of the patient, it is postulated to be the best option for cancer patients^12^. Moreover, molecular methods can efficiently detect *Toxoplasma* in fluids or tissues as it typically circulates at low concentrations, or inconstantly^13^. Very few reports are available about toxoplasmosis in cancer patients^14^. This study aimed to reveal the current trend of *T. gondii* in cancer patients utilizing serologic and molecular diagnostic methods in Sohag Governorate.

## Methods

The current research was authorized by the Ethical Institutional Review Board of the Faculty of Medicine, Sohag University, Sohag, Egypt. This study adhered to the principles outlined in the Helsinki Declaration,1964. The Medical Research Ethics Committee, affiliated with the Faculty of Medicine at Sohag University, operates under the oversight of the Office for Human Research Protections (OHRP) with the assigned identification number IRB00013006. This study was registered at Clinicaltrials.gov (ID: NCT04834076). [http://clinicaltrials.gov/ct2/show/record/ “NCT04834076]). Initial Release: 04/04/2021.

### Study design

This prospective case-control study was performed in Sohag University Hospitals, Faculty of Medicine, Sohag University, Sohag Governorate, Upper Egypt from January 2023 to December 2023. Fifty cancer patients on chemotherapy were referred to the Oncology Department, Faculty of Medicine, Sohag University, and 50 age and sex matched healthy controls were recruited from patients’ relatives for the study. Cancer patients were further categorized into 2 subgroups: solid organ malignancies and hematologic malignancies subgroups. Furthermore, any prior anti-*Toxoplasma* therapy was the exclusion criteria.

Relevant demographic data including age, and gender, were obtained for all recruited participants using a questionnaire form. Clinical data explored in patients included the type of cancer, the presence or absence of metastases, and the past history of blood transfusion. These variables were selected based on the literature. Data were obtained from the patients, physicians, and medical examination records.

Procedures were performed in the Central Research Laboratory at Sohag University and the Lab of Molecular Medical Parasitology (LMMP), Kasr Al-Ainy Faculty of Medicine, Cairo University, Egypt.

The researchers provided a comprehensive explanation of the study’s objectives and methodologies to all participants, ensuring that each participating patient provided written informed consent.

**Fig. 1:**
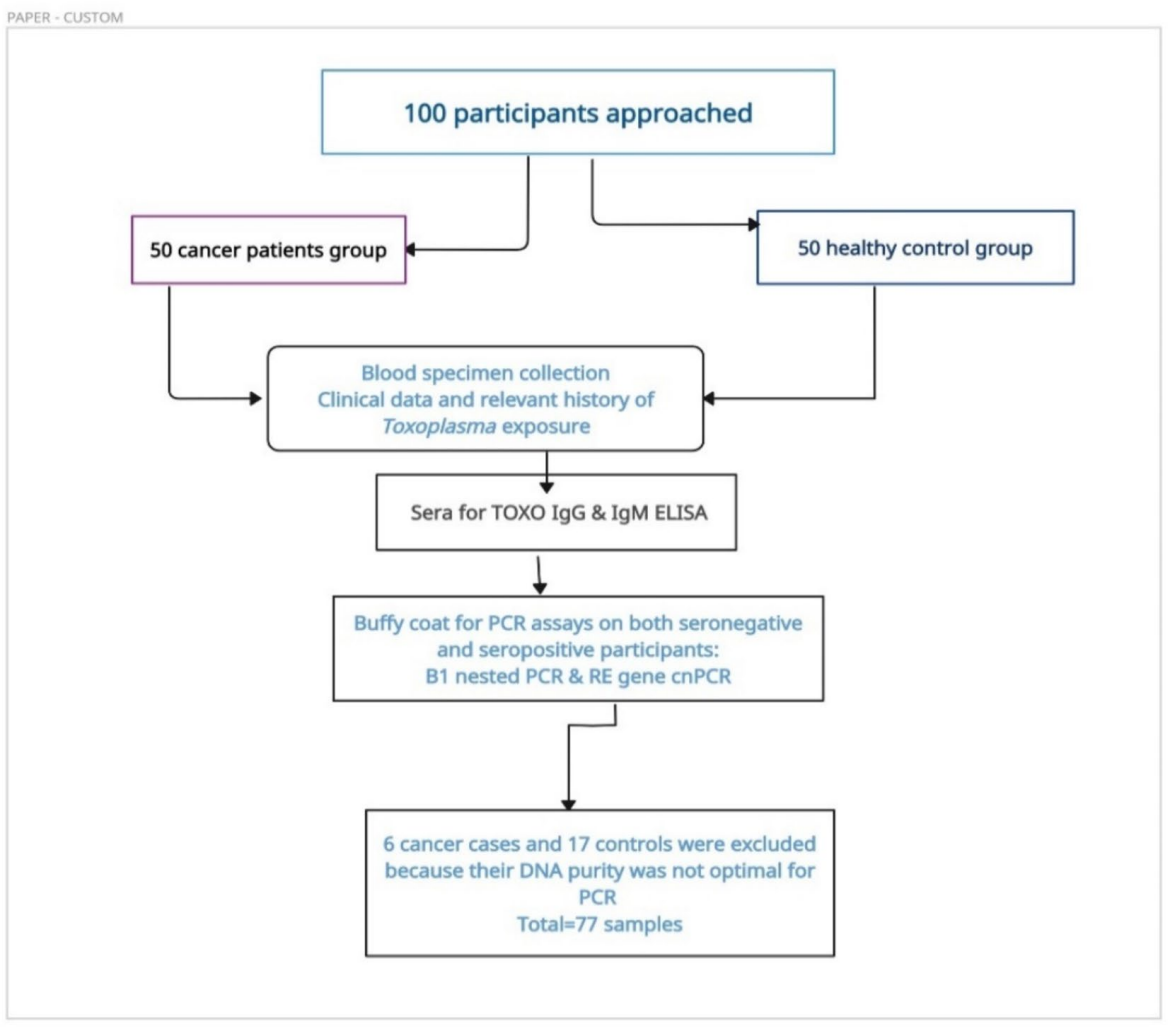
Workflow of the current study (Created by https://app.creately.com).

The workflow for this study is summarized as follows: (Fig. 1).

### Sample collection

5 ml of venous blood, from each participant, was collected via venipuncture under sterile conditions and aliquoted to sterilized (Gel Clot activator vacuum tubes) and EDTA tubes. Samples were immediately transported to the Central Research Laboratory, Faculty of Medicine, Sohag University. The blood in the Gel Clot activator vacuum tubes was left for 30 min at room temperature for clotting. Sera were separated by centrifugation at 500 g for 10 min, collected in 1.5 ml Eppendorf tubes, coded, and preserved at -20º C till the time of ELISA testing.

### Serologic diagnosis

Sera collected were screened for presence of *Toxoplasma*-specific IgG and IgM antibodies to detect their serostatus. The test was performed using the commercially available PRECHEK EIA kit Anti-TOX IgG & IgM ELISA TEST KITs (Serum/Plasma) (provided by (Precheck Bio, INC, CA, USA) for qualitative assessment of IgM and IgG. Procedural steps were performed according to the manufacturing company instructions.

Optical Densities (O.D.) were read at 450 nm using an automatic ELISA reader (THERMO ELECTRON CORPORATION Multiskan MCC 355 Microplate Reader, 51118170, Shanghai, China) within 15 min. Specimens that gave absorbances equal to or greater than the cutoff value were considered initially reactive. and positive and negative controls were included in each test.

### Molecular diagnosis

#### Genomic DNA extraction

Genomic DNA was isolated from buffy coats using Qiagen extraction blood minikit (QIAamp^®^ DNA Minikit, Hilden Germany) (Cat. No. 51104), following the manufacturer’s instructions. The DNA yield and the ratio of absorbance at 260 nm to 280 nm (A260/A280 ratio) were measured using the NanoDrop Q5000 UV-Vis Spectrophotometer (Quawell Technology, Inc, San Jose, CA, USA). The extracted DNA was stored at − 20 °C until the PCR was performed.

#### Gene targets

Two *T. gondii* multi-copy gene targets were chosen to perform the PCR assays: (1) The B1 gene, also referred to as B1 repeat, (GenBank accession no. AF179871) is a 2214 bp (bp) sequence with unknown function that is repeated 35 times in the genome of *T. gondii*. (2) The RE gene, also referred to as the 529 bp repeat, which is also of unknown function has been described in the genome of *T. gondii* in 200-300-fold repeats (GenBank accession no. AF146527.1).

Concerning the B1 gene, the external primers: B1-F1 and B1-R1 and the internal primers B1-F2 and B1-R2 were previously designed by Burg and his colleagues^15^. Refer to Table 1 which illustrates the exact sequences and the sizes of the expected amplicons.

**Table 1:** Primer sequences used in this study to amplify the B1 gene.

Primer name	Sequences	Amplicon
B1-F1	5′-GGAACTGCATCCGTTCATGAG-3′	194 bp
B1-R1	5′-TCTTTAAAGCGTTCGGGTC-3′	
B1-F2	5′-TGCATAGGTTGCAGTCACTG-3′	100 bp
B1-R2	5′-GGCGACCAATCTGCGAATACACC-3′	

The primer sequences for RE gene are shown below in Table 2 as first designed by Homan and his colleagues^16^.

**Table 2:** Primer sequences used in this study to amplify the RE gene.

Primer name	Sequences	Amplicon
TOX-4	5'CGCTGCAGGGAGGAAGACGAAAGTTG-3'	529 bp
TOX-5	5’CGCTGCAGACACAGTGCATCTGGATT-3'	

#### Nested PCR for B1 gene

The PCR Reaction of the first round was performed in final volume of 25 µl including 12.5 µl DreamTaq PCR Master Mix (2X) (Thermo scientific, U.K.), 1 µl of each primer, 0.1 µl Taq polymerase (Thermo Scientific, UK), final volume of DNA template was adjusted to 10 µl using DD H2O. The amplicon of the first round was utilized as a DNA template for the second round PCR reaction. The final volume was also 25 µl, containing 12.5 µl PCR Master Mix 2X (Thermo Scientific™), 1 µl of each primer, 0.1 µl Taq polymerase (Thermo Scientific, UK), 8.4 µl dd H2O and 2 µl of DNA template.

A 96-well thermocycler (Thermo Scientific Arktik™TCA4848) was used for the PCR amplification process. The amplifications were made in two sequential steps. The conditions of the two rounds are illustrated in Table 3.

**Table 3:** Thermal cycling conditions used in nested PCR assay (B1 gene).

	Step 1-B1	Cycles no	Step 2-B1	Cycles no
Initial denaturation	94ºC 3′		94ºC 3′	
Denaturation	94ºC 60′′	30X	94ºC 60′′	35x
Annealing	55ºC 60′′		60ºC 60′′	
Extension	72ºC 60′′		72ºC 60′′	
Final extension	72ºC 7′		72ºC 7′	

Each PCR run included a positive control (pure tachyzoites of RH strain preserved in PBS at -20º C) kindly provided by Professor Mona Mohamed Tolba, Parasitology Department, Medical Research Institute, Alexandria University, Egypt and Professor Eman El-Wakil, Parasitology Department, TRBI, Egypt. Furthermore, DNase-free water was used as a negative control to exclude contamination in the reaction.

#### PCR for the RE gene

The PCR Reaction was performed in 25 µl total volume containing 12.5 µl DreamTaq PCR Master Mix (2X) (Thermo scientific, U.K), 1 µl of each primer, 0.1 µl Taq polymerase (Thermo scientific, UK), final volume of DNA template was adjusted to 10 µl using DD H2O. The conditions of the optimized PCR experiment are illustrated in Table 4.

**Table 4:** Thermal cycling conditions used in PCR assay (RE gene).

	RE gene	Cycle no
Initial denaturation	94ºC 5′	
Denaturation	94ºC 60′′	35x
Annealing	65ºC 60′′	
Extension	72ºC 60′′	
Final extension	72ºC 7′	

After thermal cycles, PCR products were electrophoresed in 1.5% agarose gel and stained with Ethidium bromide (EtBr) in horizontal gel electrophoresis. DNA fragments were visualized under ultraviolet (UV) transilluminator. The size of DNA fragments was determined on comparison with 100-bp ladder.

### Statistical analyses

Data was analyzed using STATA version 17.0 (Stata Statistical Software: Release 17.0 College Station, TX: Stata Corp LP.). Data were expressed as mean ± SD and counts or percentages where appropriate. The Mann–Whitney test was used for non-parametric data and the χ2 test was used for categorical variables. The Fisher’s exact test was used when the expected cell count deviation was < 5. ROC curve was depicted to explore the diagnostic performance of RE gene PCR, analysed as area under the curve (AUC), standard error (SE) and 95% CI. Validity statistics (sensitivity, specificity, positive and negative predictive value –PPV & NPV-) were calculated. Logistic regression analysis was calculated to investigate the independent association between infection parameters and cancer (Odds Ratio -OR-, 95% confidence interval − 95% CI- and p-value-). A p-value of < 0.05 was considered statistically significant.

## Results

### Clinical and demographic data of the study groups

The age range of cancer patients spanned from 3 to 76 years, with a mean age of 37.46 ± 18.57 years and IQR (40.5). This was comparable to the age range observed in the healthy individuals, which was 4 to 69 years, with a mean age of 37.0 ± 14.72 years and IQR (37). Cancer patients included 28 males (56%) while the control group included 27 males (54%). Both groups were comparable and matched for age and sex (Table 5).

**Table 5:** Differences in baseline characteristics among the studied groups.

	Controls (n = 50)	Cases (n = 50)	P-value
Age			
Mean ± SD	37.00 ± 14.7	37.46 ± 18.6	= 0.158*
Median (IQR)	37 (23)	40.5 (26)	
Sex			
Female	23 (46%)	22 (44%)	= 0.841*
Male	27 (54%)	28 (56%)	

*Mann Whitney U-test was used to compare the median difference between groups.

**Chi-square test was used to compare the proportion difference between groups.

### Serologic findings

The overall seroprevalence of *T. gondii* IgG in all the study participants was high (58%). However, *T. gondii* IgM was detected in only 9 study participants (9%). This study compared the seropositivity of *Toxoplasma* IgG and IgM in cancer cases versus the control group and no differences of statistical significance was detected (*p* = 0.25 & 0.49, respectively) as shown in Table 6.

**Table 6:** Distribution of *T. Gondii* IgG and IgM among cancer cases and control group.

	Cancer group		Control group		*P* value	OR
	Seropositive	Total tested	Seropositive	Total tested		
*T. gondii* IgG	30 (60%)	50 (100%)	28 (56%)	50 (100%)	0.25	1.15 (0.52–2.57)
*T. gondii* IgM	6 (12%)	50 (100%)	3 (6%)	50 (100%)	0.49	2.11 (0.50–8.97)

*Regression model included age, sex residence as a-priori factors in addition to *T. gondii* IgG and IgM.

**Fig. 2:**
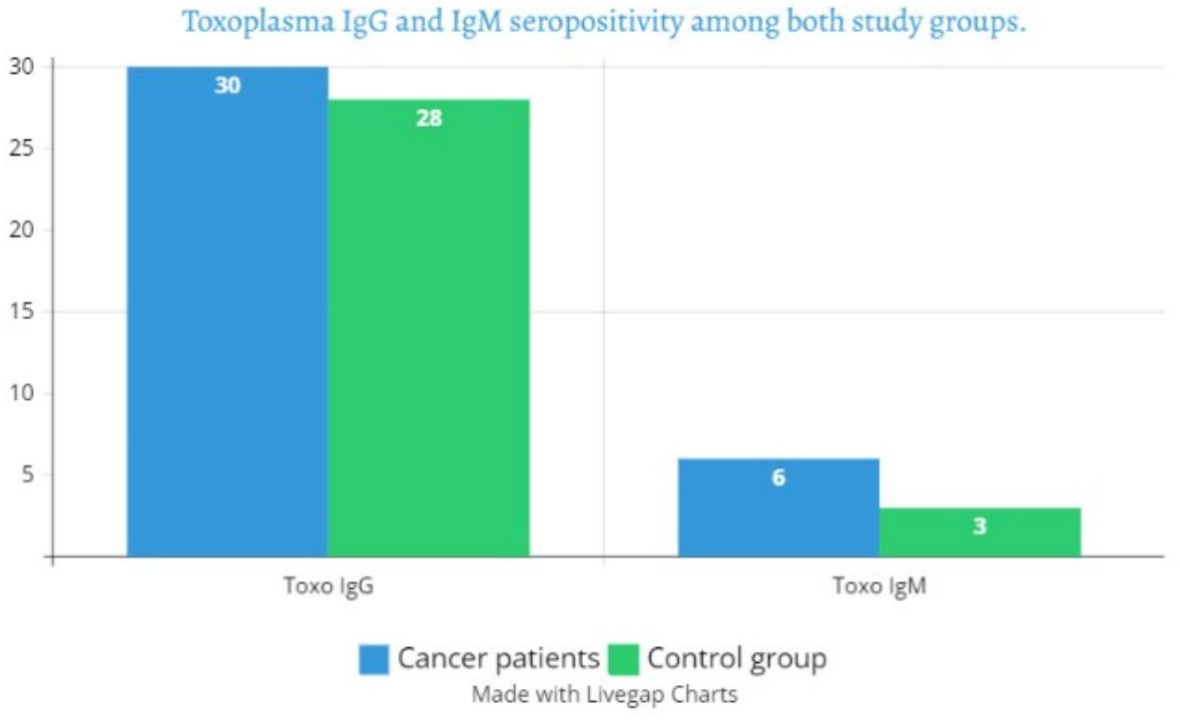
Bar graph showing the frequencies of *Toxoplasma* IgG and IgM among both study groups.

IgG seropositivity was detected in a nearly same fashion in cancer and control groups (30% and 28%, respectively). However, cancer cases showed double infection risk by *Toxoplasma* IgM (12% versus 6%, respectively). The results are depicted in the following bar graph (Fig. 2).

The cancer group was further categorized into two subgroups: hematologic and solid cancer groups. Patients diagnosed with hematologic malignancies represented (56%) while (44%) had solid cancers.

This study revealed that patients with solid organ cancers exhibited a greater rate of IgG seropositivity compared to those with hematologic tumors (77.27% and 46.43%, respectively). This difference was found to be statistically significant (*p* = 0.03). Results are listed in Table 7.

**Table 7:** *T. Gondii* IgG and IgM seropositivity among hematologic and solid cancer groups.

		Hematologic cancers	Solid cancers	*P* value
*T. gondii* IgG	Negative	15 (53.57%)	5 (22.73%)	0.03
	Positive	13 (46.43%)	17 (77.27%)	
*T. gondii* IgM	Negative	23 (82.14%)	21 (95.45%)	0.21**
	Positive	5 (17.86%)	1 (4.55%)	

*P* value was calculated using the Chi square test, **P value was calculated using fisher exact test.

*P* value in bold is < 0.05 (statistically significant).

Table 8 illustrates the different serologic combinations of *T. gondii* IgG and IgM among both study groups. The highest serologic combination “IgM-, IgG+” accounted for 52% in both cancer and control groups. However, only two cancer cases and one healthy individual were “IgM+, IgG-”. No differences of statistical significance were found.

**Table 8:** Distribution of the studied population by ELISA in cancer cases and controls.

ELISA results	Cancer cases	Controls	OR (95% CI)	*P* value
IgM − , IgG −	18 (36.00%)	21 (42.00%)	0.78 (0.32:1.87)	0.54
IgM − , IgG +	26 (52.00%)	26 (52.00%)	(0.42:2.36)	1.00
IgM + , IgG −	2 (4.00%)	1 (2.00%)	2.04 (0.10:122.86)	0.56
IgM + , IgG +	4 (8.00%)	2 (4.00%)	2.09 (0.28:23.94)	0.40

*P* value was calculated using the Chi square test.

*P* < 0.05 is statistically significant.

*Regression model included age, sex residence as a-priori factors in addition to *T. gondii* IgG and IgM.

The distribution of the seroprevalence of *T. gondii* IgG according to the clinical diagnosis of cancer is presented in Table 9. Lung and colon cancers (100%) exhibited the highest prevalences of *T. gondii* followed by brain and hepatocellular carcinoma (75%). There was no statistical significance. Moreover, Hodgkin’s and non-Hodgkin’s lymphomas had (55.56%) and (71.43%) seroprevalences, respectively. However, no statistical significance was found.

**Table 9:** The distribution of *Toxoplasma* IgG seropositivity among various hematologic and solid malignancies.

Cancer type	IgG seropositivity		P value
	Negative	Positive	
Hematologic cancers			
Acute lymphoblastic leukaemia	7 (77.78%)	2 (22.22%)	0.28
Non-Hodgkin lymphoma	4 (44.44%)	5 (55.56%)	
Hodgkin lymphoma	2 (28.57%)	5 (71.43%)	
Chronic lymphoblastic leukaemia	1 (50.00%)	1 (50.00%)	
Acute myeloid leukaemia	1 (100%)	0	
Solid cancers			
Breast cancer	3 (27.27%)	8 (72.73%)	0.90
Brain cancer	1 (25.00%)	3 (75.00%)	
Hepatocellular carcinoma	1 (25.00%)	3 (75.00%)	
Lung cancer	0	2 (100%)	
Colon cancer	0	1 (100%)	

*P* value was calculated using the Chi square test.

*P* < 0.05 is statistically significant.

As demonstrated in Table 10, *Toxoplasma* IgM seropositivity was reported only in one case of the solid cancers subgroup which was a case of lung cancer (50%). Moreover, there was a statistical significance when comparing the solid cancers subgroup (*P* = 0.03).

**Table 10:** The distribution of *Toxoplasma* IgM seropositivity among various hematologic and solid malignancies.

Cancer type	IgM seropositivity		P value
	Negative	Positive	
Hematologic cancer			
Acute lymphoblastic leukemia	8 (88.89%)	1 (11.11%)	
Non-Hodgkin lymphoma	8 (88.89%)	1 (11.11%)	
Hodgkin lymphoma	5 (71.43%)	2 (28.57%)	0.60
Chronic lymphoblastic leukemia	1 (50.00%)	1 (50.00%)	
Acute myeloid leukemia	1 (100%)	0	
Solid cancer			
Breast cancer	11 (100%)	0	
Brain cancer	4 (100%)	0	
Hepatocellular carcinoma	4 (100%)	0	**0.03**
Lung cancer	1 (50.00%)	1 (50.00%)	
Colon cancer	1 (100%)	0	

*P* value was calculated using the Chi square test.

*P* < 0.05 is statistically significant (in bold).

### Molecular findings

**Fig. 3:**
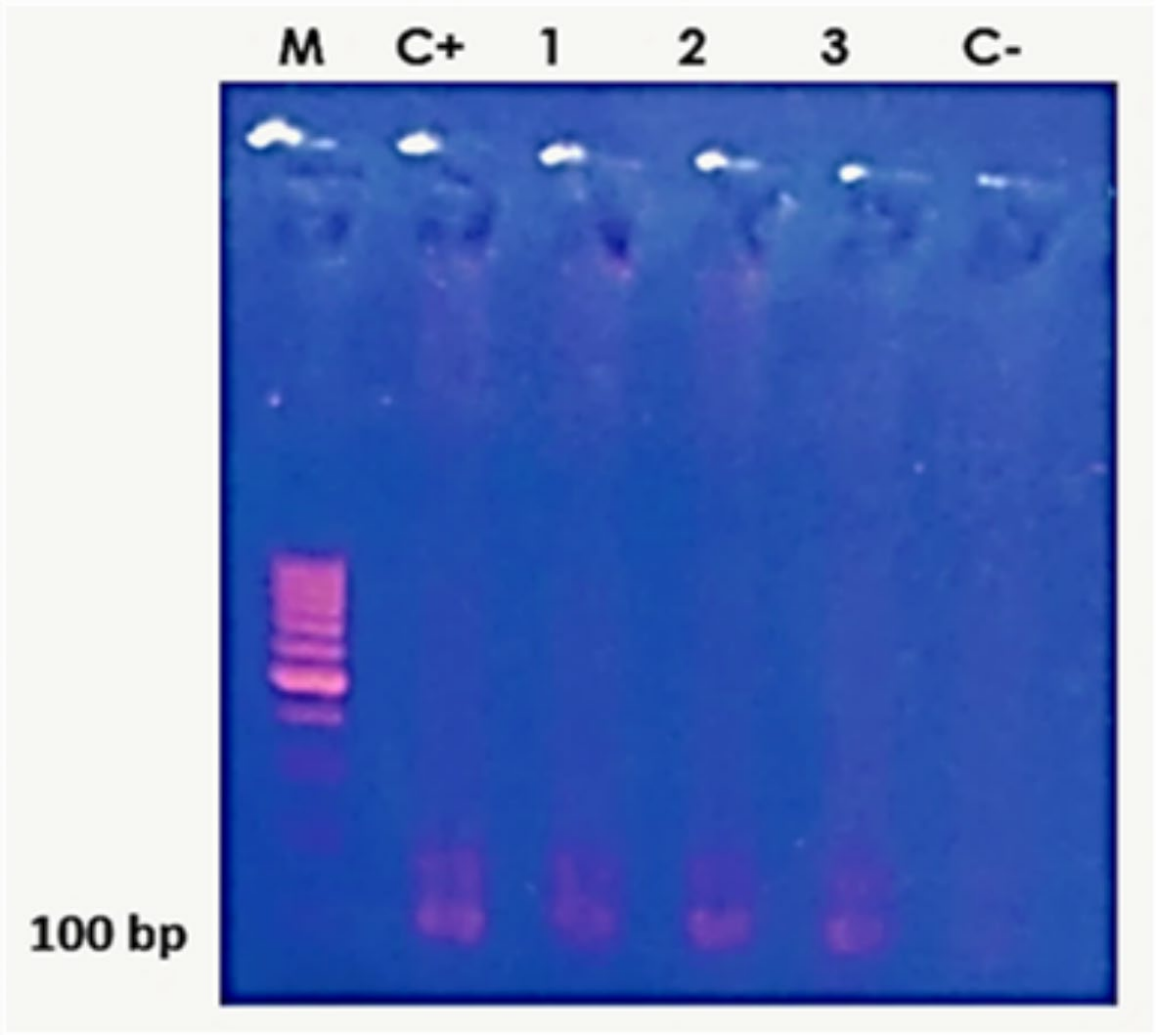
Results of the nested PCR using *Toxoplasma* B1 gene: Lane C + shows RH strain as a positive control. Lanes 1, 2 and 3 show the second nested PCR amplification of the B1 gene. Lane C- is the negative control. M is the 100 bp molecular weight marker.

The DNA of the 100 samples, positive and negative for anti-*Toxoplasma* antibodies, was extracted. After DNA purification, out of the 100 samples, 6 samples from cancer cases and 17 samples from controls (total = 23 samples) were excluded from the molecular experiments because their DNA concentration and/or purity were not optimal. This study found that only 4 cancer cases were positive by both PCR experiments. None of the samples of the healthy controls could amplify *Toxoplasma* B1 nor RE genes. According to our nested-PCR assay on buffy coat samples from the cancer patients and the non-cancerous control group, only 4 (9%) out of 44 cancer patients were positive regarding *T. gondii* B1 gene amplification. Additionally, none out of the samples of the 33 healthy individuals showed a specific 100 bp DNA band in 1.5% agarose gel (Fig. 3).

**Fig. 4:**
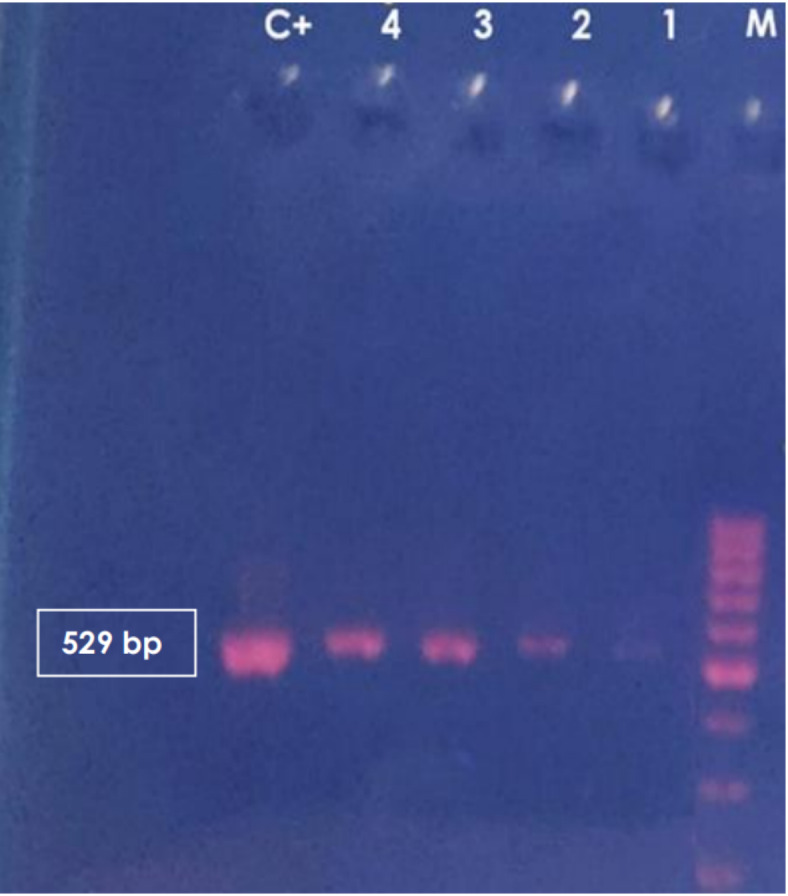
PCR products of *T. gondii* from the buffy coats of 4 cancer patients. M: 100 bp molecular weight marker; C+: positive control; lanes 1,2,3 and 4: PCR products of RE gene.

To amplify the 529 bp fragment, PCR was carried out on 77 buffy coat samples of the study subjects regardless of the presence or absence of anti-*T. gondii* antibodies using the RE gene marker. From a total of 77 buffy coat samples, from the cancer patients and healthy controls, 4 (9%) samples contained *T. gondii* DNA and showed a 529 bp band in 1.5% agarose gel (Fig. 4). The 4 samples that successfully amplified RE genes were cancer patients and none of the healthy controls’ samples could amplify the gene.

The agreement between PCR and nested PCR was calculated using the Cohen kappa coefficient (κ). The analyzed results were interpreted considering the ranges published by Landis and Koch^17^. The analysis of the obtained results showed that the same 4 samples were positive by both PCR and nested PCR. Cohen kappa coefficient κ was 1 indicating perfect agreement.

Table 10 depicts the diagnostic parameters of PCR (RE gene) versus nested PCR assay. The obtained results showed that the RE gene-PCR had a sensitivity, specificity, PPV, and NPV of 100% (Table 11).

**Table 11:** Diagnostic performance parameters of the RE gene PCR.

		Nested PCR (B1 gene)		Total	AUC	Sensitivity (%)	Specificity (%)	PPV (%)	NPV (%)
		Positive	Negative						
PCR (RE gene)	Positive	4 (100%)	0 (0%)	4	1	100%	100%	100%	100%
	Negative	0 (0%)	4 (100%)	4					
	Total	4	4	8					

**Fig. 5:**
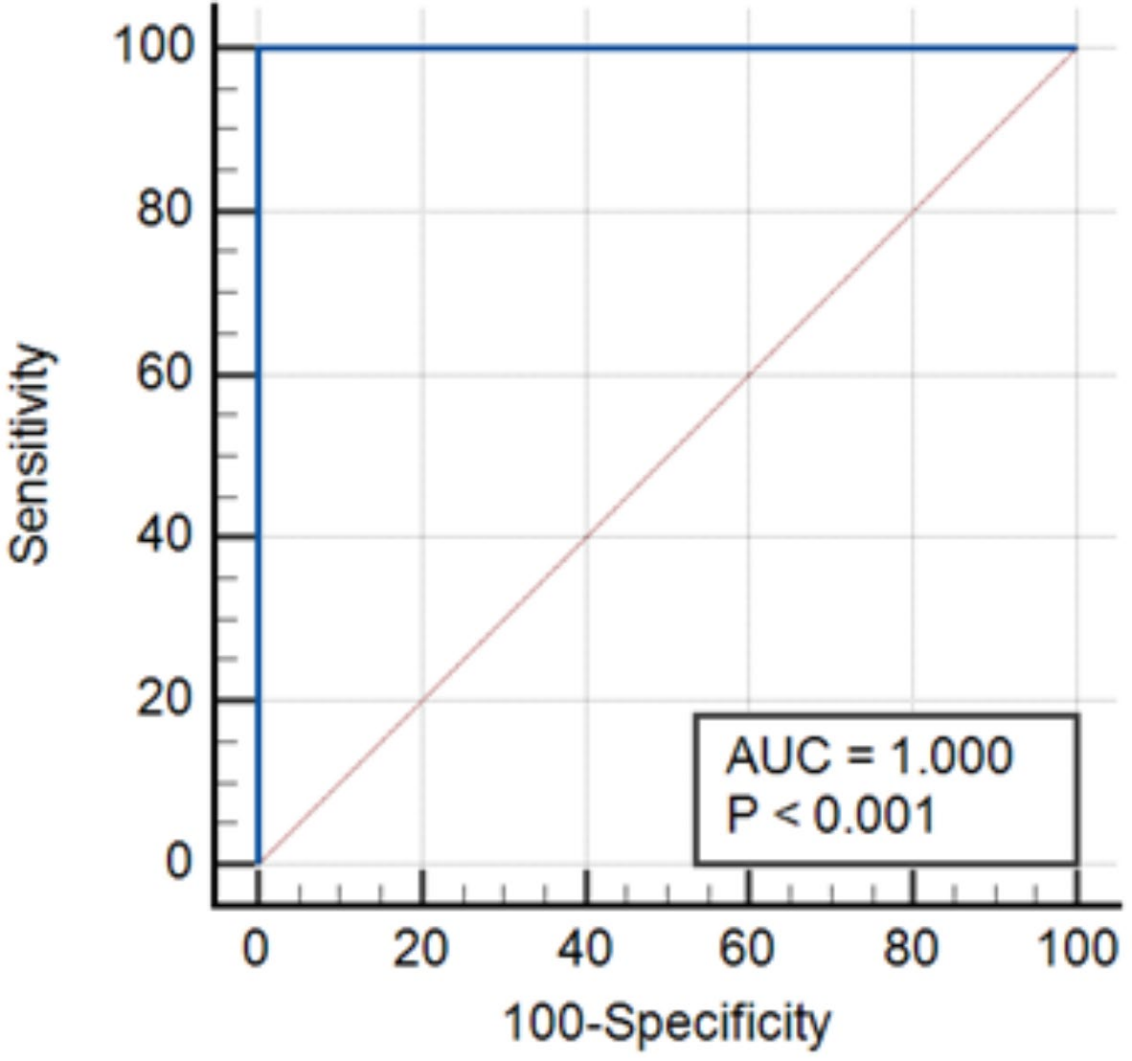
Area under the ROC curve of the PCR (RE gene) versus the nested PCR (B1 gene) as the gold standard diagnostic test.

The ROC curve analysis was conducted to estimate the diagnostic performance of PCR (RE gene). The area under the curve (AUC) was calculated and it was 1.0, thereby indicating a perfect test (i.e., 100% sensitive) (Fig. 5).

Out of the four PCR-positive samples, 2 cases were solid cancers (hepatocellular carcinoma and brain cancer). In addition, 2 cases were diagnosed with HL. One HL patient was seronegative for both *Toxoplasma* IgG and IgM. The other 3 PCR-positive patients were “IgM-, IgG+” (Table 12).

**Table 12:** The serostatus of the PCR-positive cancer cases according to their clinical diagnosis.

PCR + ve cancer cases	IgM-, IgG-	IgM-, IgG +
Hodgkin’s lymphoma (HL)	1(25%)	1(25%)
Hepatocellular carcinoma (HCC)	0	1(25%)
Brain cancer	0	1(25%)

The RE PCR and B1-nested PCR produced positive results (the presence of *T. gondii* DNA) for 3 of the 43 IgM-, IgG + seropositive samples. Out of 25 seronegative samples (IgM−, IgG−), one sample was successfully amplified. However, none of the (IgM+, IgG-) and (IgM+, IgG+) were positive by PCR. The positive samples were the same in the 2 different PCR techniques (Table 13).

**Table 13:** Comparative results of the PCR assays on “IgM+, IgG+, 10 IgM−, IgG + and IgM−, IgG−“samples.

	PCR-negative (n = 73)	PCR-positive (n = 4)
IgM − , IgG −	24 (32.88%)	1 (25.00%)
IgM − , IgG +	40 (54.79%)	3 (75.00%)
IgM + , IgG −	3 (4.11%)	0
IgM + , IgG +	6 (8.22%)	0

## Discussion

The escalating incidence of cancer cases has highlighted the significance of toxoplasmosis. It ranks as the third leading cause of mortality in immunocompromised patients following *Pneumocystis* and *Cryptosporidium*. In spite of its crucial role, it failed to receive the attention it deserves^18^. Clinical and epidemiological studies have shown evidence of a possible link between parasitic protozoan infections and cancer. *T. gondii* is associated with leukemia and lymphoma^19^. There is no data about the current trend of *T. gondii* infection in our governorate. Hence, the current case–control study is the first to determine the sero-epidemiology and molecular detection of *T. gondii* in cancer patients from Sohag Governorate, Upper Egypt compared to healthy controls.

The overall seroprevalence of *T. gondii* IgG in all the study participants was high (58%). A recent review by Rouatbi et al*.*^20^ discussed prevalence rates in North Africa and reported that the overall seroprevalence of *T. gondii* in in Egyptians is reaching 59.6% which is consistent with the results obtained by this study. Lower prevalence rates were reported in aborted women (35.6%) and primigravidae pregnant women from Sohag governorate (47.1%)^21,22^. The current study revealed that *T. gondii* IgG was slightly higher in cancer patients than the control group (60% and 56%, respectively). Although, the IgM prevalence was found to be two folds in cancer patients than healthy controls (12% versus 6%, respectively), it was insignificant. Yet, cancer patients are still more prone to toxoplasmosis (OR = 2). This could be justified that reactivation of infection, and the risk of reinfection are seemingly similar in all individuals. The presence of specific IgG and absence of IgM strongly points to a prior infection. However, the IgM can be occasionally evanescent or even negative^23^.

To monitor any possible rise in IgG levels, it is recommended to perform a second serology 3 weeks later. Stable IgG levels denote chronic toxoplasmosis. A significant increase in IgG levels prompts performing determination of IgG avidity. Reinfection or reactivation is strongly suspected in cases of high IgG avidity. Serological follow-up is unnecessary if this situation occurs in an immunocompetent subject^24^. It is challenging to make a definitive serologic diagnosis of acute infection from a single serum sample because IgM antibodies can last for months, and the avidity index may stay low for several months^25^, thus definitive diagnosis requires the sequential appearance of specific IgM and IgG antibodies in the same sample. We could not do this due to financial constraints. It is inevitable to choose the right technique at the appropriate cost. Confirmation methods are mostly inhouse, complex, or costly methods, such as dye test, IFAT, and immunoblot. Furthermore, they should be utilized exclusively in reference laboratories^24^. It is worth to take into consideration that seropositivity is not always indicative of latent infection, because cancer patients may frequently receive blood transfusions, that could lead to passively transferred antibodies^26^.

Few recent studies from Egypt reported various seropositivity rates of *T. gondii* in patients suffering from various malignancies. For instance, Abdel Malek et al*.* and Saad et al*.*^27,28^ had detected *T. gondii* IgG in 40%, and 20%, and IgM in 5.8%, and 4% cancer patients, respectively. This was lower than our results. Moreover, Saad et al*.* reported that seropositive cases for anti-*Toxoplasma* IgM and IgG accounted for (4.3%) & (56.5%) in cancer group^29^. Similar seroprevalence rates of anti-*T. gondii* IgG in Egyptian cancer patients from different governorates were reported (55.8%, 61.1%, 62.5%, 66.7% and 67.5%)^27,29–32^. Recent reports’ data, both national and regional, warrant cautious interpretation due to limited sample sizes and inadequate information on population characteristics and pertinent *Toxoplasma* risk factors.

Furthermore, this disparity is influenced by the various geographical and environmental factors which facilitate the transmission of infection. Additionally, there is a noticeable discrepancy in the prevalence of toxoplasmosis between past and modern studies conducted in Egypt. This variation emphasised the present escalating baseline rate of *T. gondii* seropositivity and indicated the heightened pace of infection transmission throughout the last two decades. In 1991, Khalil and colleagues documented the existence of anti-*Toxoplasma* antibodies in 36% of lung cancer patients^33^. Many similar studies were conducted worldwide with variable seroprevalence rates. For instance, anti-*T. gondii* IgG was reported in 60% in Turkish cancer patients which was consistent with our study results^34^. Moreover, anti-*T. gondii* antibodies (IgG) were detected in 82.1% of Iraqi cancer patients^35^. This was significantly higher than this study results. Conversely, lower prevalences of anti-*T. gondii* IgG antibodies were reported from China, Iran, Saudia Arabia and Jordan (35.56%, 36.6%, 30.6% and 39.5%, respectively)^19,26,36,37^.

The occurrence of IgM antibodies solely (IgM positive/IgG negative) is seldom observed because of the brief interval between their appearance. This coincides with the current results, which indicate that IgM was identified exclusively in 3% of the samples, while IgM and IgG were detected concurrently in 6% of the samples. This study revealed that patients with solid organ malignancies showed a larger proportion of IgG seropositivity compared to those with hematologic tumours (77.27% and 46.43%, respectively). This difference was determined to be statistically significant (*P* = 0.03). The findings also demonstrated that *Toxoplasma* IgM had a prevalence of (17.86%) in haematological malignancies and (4.55%) in solid organ cancers. This difference was determined to be statistically non-significant. This agreed with Abdel Malek et al*.*^28^ who stated that patients with solid organ tumors had a two times higher prevalence rate of toxoplasmosis (24%) compared to hematological malignancies (12%), and this difference was statistically insignificant (*P* = 0.06). Conversely, Ali et al*.*^31^ reported that patients with hematological malignancies had greater IgG seropositivity compared to those with solid organ tumours (40% and 26.7% respectively), with a statistically significant difference (*P* = 0.002). Furthermore, a recent Iranian study revealed that a greater occurrence of anti-*Toxoplasma* IgG antibodies in patients diagnosed with lymphoma and leukaemia (*P* = 0.001) compared to those with other solid cancerous masses^18^. Nevertheless, two explanations can elucidate our findings. Firstly, the majority of patients with solid tumours have compromised cellular immunity. Their classification is that of high-risk neutropenic patients. Secondly, corticosteroids are the cornerstone in the majority of chemotherapy agents prescribed to patients with haematological malignancies. The greatest frequency of *T. gondii* IgG was found in 100% of lung and colon malignancies, followed by brain and hepatocellular carcinomas (75%). This was relatively in concordance with an earlier lung cancer patients had the greatest rate of *T. gondii* infection (60.94%), followed by cervical cancer patients (50.00%) and brain cancer patients (42.31%)^36^. Inconsistently, Iraqi breast and colon cancer patients had higher anti-*T. gondii* IgG seropositivity (77.50%, 77.42%) than the control group (20.54%)^38^. However,^31^ reported that all (100%) stomach and bone cancers were positive for *Toxoplasma* IgG. This is somewhat consistent with the findings of Mostafa et al.^27^, who found that the highest IgG positivity was found in breast cancer (96.1%), followed by bone carcinoma (80%), brain tumour (69.2%), and liver tumour (35%). A recent meta-analysis was conducted to assess the potential the association between *T. gondii* infection/exposure and the likelihood of the brain tumours development. The pooled analysis revealed an overall (OR) of 1.96 (95% CI, 1.37–2.80), suggesting a substantial higher risk of brain tumours linked to *T. gondii* infection /exposure^39^.

The current research found that *Toxoplasma* IgM seropositivity was the highest equally in lung cancer and chronic lymphoblastic leukemia (50%), followed by Hodgkin’s lymphoma (28%). The high seroprevalence in leukemic patients agreed with the results of an evidence based meta-analysis (OR = 3.05; 95%CI = 1.83–5.08)^40^. Contrary to this, the authors of a Chinese study reported that the positivity of *Toxoplasma*-IgM antibodies was the highest in intracranial malignancies (7.69%), followed by lymphoma (4.76%), hepatocellular carcinoma (3.64) and lastly, breast cancer (3.57)^41^. A relatively lower prevalence was reported. (28.9%) of CCL patients were positive for anti-*T. gondii* IgM antibodies in a case–control study conducted on leukemic Chinese children^42^. Furthermore, Kalantari and his colleagues^19^ stated that anti–*T. gondii* IgM was not detected in paediatric hematologic malignancies. In the current study, it was found that the highest serologic combination was “IgM-, IgG + ” which accounted for 52% in both cancer and control groups. However, only two cancer cases and one healthy individual were “IgM + , IgG-”. No differences of statistical significance were found. This can be clarified that most Egyptians; cancer patients and the healthy individuals had a past infection. In other words, they are chronically infected, and they are at risk of reactivation.

Whilst the objective of the search for *Toxoplasma* IgM antibodies was to confirm the acute infection, it soon became evident that IgM can persist for several months or even years. A recent excellent review provided a comprehensive analysis of multiple mechanisms that may enhance our comprehension of the “chronic IgM” phenomenon. One of them is that micro-reactivation (excystment)– (encystment) cycles of *T. gondii* can consistently produce low levels of IgM for extended durations. Tested by both non reference laboratories (NRLs) and reference laboratories, *Toxoplasma* IgM alone is unable to differentiate between a freshly contracted infection and a chronic one^43^. A complete serologic panel should be performed at the reference laboratory as mandated by the FDA for an accurate interpretation for all *Toxoplasma* IgM specimens identified as positive by NRLs^44^. Occasionally, serology may not be sufficient to detect active infection in individuals with impaired immunity. Multiple factors may hinder the generation of specific anti-*Toxoplasma* antibodies when undergoing antineoplastic therapy. This may result in a reduction of the titre to negligible levels. In other circumstances, the antibody response may be delayed for a duration beyond 4 weeks. These parameters may account for the lack or delay of a recent increase in IgM production in cancer patients recruited in the current study^45^.

Innovations in molecular diagnostic techniques during the past 30 years have completely transformed the diagnosis of parasite diseases. Owing to their exceptional sensitivity and specificity, they are increasingly recommended as a crucial diagnostic tool for congenital toxoplasmosis, toxoplasmosis in immunocompromised persons, and ocular toxoplasmosis^46^. They are independent of the immune response, so they may facilitate more accurate diagnosis. Hence, it is highly advised to use PCR assays to detect *T. gondii* genomic DNA in blood or bodily fluids as a complementary diagnostic approach, particularly in immunocompromised individuals^47^. Even so, in developing nations, including Egypt, molecular techniques are not frequently used in clinical settings for routine diagnosis and monitoring due to their high cost and time-consuming nature. The B1 gene (30–35 copies/genome) and the Rep529 repetitive elements (200–300 copies/genome) are multi-copy, highly conserved genes in the *T. gondii* genome estimated to have higher analytical sensitivity than single copy genes for detecting *T. gondii* DNA^48^. Therefore, in the present study, PCR assays relying on the *T. gondii* B1 gene and the RE 529-bp region were chosen in particular because to their established trustworthiness in recent literature. These are some examples^49–56^. The current study performed molecular detection of *Toxoplasma* DNA on buffy coat extracts from 44 cancer cases and 33 controls regardless of their serostatus. Two PCR techniques were performed: nested PCR and PCR for detection of the B1 gene and the rep-529 fragment (RE gene), respectively.

Our results revealed that only 4 samples of cancer patients (5.19%) could successfully amplify both the B1 gene and the 529 bp fragment. However, all the controls were *Toxoplasma*-PCR negative. Generally, the detection of *T. gondii* in clinical samples confirms its existence which can be due to initial, reactivated, or persistent infection^1^. Despite finding of *Toxoplasma* DNA in blood indicates parasitemia, its clinical value for persons with compromised immune systems remains questionable^57^. Numerous studies have shown the inadequate sensitivity of PCR techniques using peripheral blood, presumably due to low parasite concentrations^58^. The present study suggested that sensitivity of the RE-based PCR experiment was equivalent to the B1 gene PCR. This was unexpectedly different from a large body of evidence which supports the superiority and accuracy of the RE segment over the B1 gene. This can be clarified by the fact that the B1 PCR is more specific but less sensitive than the RE PCR due to the difference in the number of their copies in *T. gondii* genome. When the sensitivity of the PCR targeting the B1 gene was enhanced by performing nested PCR, both had the same sensitivity and specificity. Nevertheless, participants exhibited a low positivity rate that did not correlate substantially to their serological profile.

In 2010, it was reported that the rep 529 element was absent in 4.8% of human *T. gondii*-positive samples. The data supported that the B1 gene may be the target of choice^59^. Thereafter, similar results were stated^60^. Despite this, a recent work has shown that the effectiveness of Rep 529 qPCR is not influenced by the genotype of *T. gondii* isolates and truly, it outperforms B1 qPCR accordingly^60^. The low PCR yield in the present work was similar to what was found by^55,61–63^ (8.3%, 10%, 11% and 11.1%, respectively). Our results strongly go in agreement with the results reported previously in 2021. The authors stated that (5.43%) samples contained *T. gondii* DNA and showed the expected 529 bp band^18^. Additionally, a 3-year retrospective study was carried out in 15 reference laboratories from the network of the French National Reference Centre for Toxoplasmosis. The predominant source of positive samples was blood, accounting for 55% of the cases. This was followed by CSF (17%), AF (16%), BAL (5%), and different tissue biopsy samples (7%). *T. gondii* DNA detection in blood was whether DNA extraction was performed using whole blood or the leukocyte fraction^49^. On the contrary, lower detection rates (3.55%) and (1%) were previously reported in China and Iran^41,64^. The diversity in outcomes may be attributed to different genotypes of *T. gondii*, particularly highly virulent strains along with variations of parasitemia. In Egypt, a paucity of studies has been performed to molecularly diagnose toxoplasmosis in cancer patients.

Recently, *Toxoplasma* DNA was revealed in 32.5% of children with hematologic malignancies which was higher than our result^32^. As a matter of fact, this tremendous heterogenicity in *Toxoplasma* PCR detection rates could be attributed to the lack of standardization. In other words, the utilization of different DNA purification methods and Kits, gene targets, primer sets, PCR protocols, sample types, sample size and lastly different study subjects. The RE-PCR and B1-nested PCR produced positive results for 3 of the 43 (IgM-, IgG +) seropositive samples. Out of 25 seronegative samples (IgM − , IgG −), one sample was successfully amplified. However, none of the (IgM + , IgG-) and (IgM + , IgG +) were positive by PCR. In other words, the present study revealed that 3 (75%) out of the 4 cancer cases positive by PCR were (IgG + , IgM-), and one case (25%) was (IgM-, IgG-) seronegative. The presence of a positive PCR in a patient with negative serology is a crucial indication that a seronegative result obtained by ELISA does not provide a definitive negative result for toxoplasmosis. Therefore, it is necessary to conduct more sensitive supplemental tests^52^. The presence of a positive PCR in a patient with negative serology is a crucial indication that a seronegative result obtained by ELISA does not provide a definitive negative result for toxoplasmosis. Therefore, it is necessary to conduct more sensitive supplemental tests. Inability to find *Toxoplasma* DNA in a significant proportion of seropositive patients was documented in various population cohorts^32^. This may be elucidated by the limited quantity of DNA found in blood samples^65^. The successful amplification of *T. gondii* DNA in blood samples appears to be influenced by the timing of sampling following infection due to the transitory nature of parasitaemia^45^.

Further variables contributing to negative PCR results are the limited sample size used for DNA extraction in relation to the overall blood volume in the human body, and the presence of certain PCR inhibitors, namely heme, haemoglobin, and lactoferrin^66^. In addition, the analysis should be performed in multiplicate^65^. Due to the restricted financial resources of this study, the PCR assays were performed in uniplicates. This may be a factor of getting this low PCR detection rate. Prior studies have indicated that PCR sensitivity varies between 12 and 100% for CSF and between 15 and 80% for blood. The specificity and PPV of PCR can reach up to 100%. A negative PCR result does not, however, formally rule out reactivation^67^. Diagnostics of toxoplasmosis in individuals with impaired immune systems should be conducted via algorithms including a combination of molecular, serologic, clinical, and radiologic findings^68^. The IgG-positive result indicates that these three patients are possibly experiencing chronic toxoplasmosis. PCR-positivity suggested that treatment with immunosuppressive drugs rendered them more prone to reactivation. The 4^th^ patient tested positive for PCR and obtained negative results for anti-*Toxoplasma* IgM and IgG antibody tests. The above findings imply evidence of a novel infection. The negative ELISA test could be due to the immunosuppressive treatment. The diminished detection rate cannot definitively rule out recent infection and could be attributed to a limited quantity of parasites in the bloodstream, a low concentration of parasite DNA in clinical samples relative to the whole blood volume in the human body, or a brief duration of parasitemia. The seronegative cases, in which the B1 gene was definitively detected by PCR, could indicate the existence of circulating *Toxoplasma* tachyzoites that are not correlated with antibodies. The hypothesised interpretation is that the infection may have occurred very recently during serological testing, resulting in a minimal production of detectable antibodies by serology^69^. In accordance with our findings, PCR indicated that 5.05% of cancer patients tested positive for the B1 gene of *T. gondii*. The anti-*Toxoplasma* IgM antibody test yielded negative results for all five positive cases. Out of the five positive cases, four contained anti-*Toxoplasma* IgG antibodies, while one tested negative for ELISA IgG antibodies^45^. By contrast, *Toxoplasma* DNA was found in 9 out of 9 samples of IgM + /IgG + and 10 out of 255 samples of IgM − /IgG + ^18^. Furthermore, PCR screening revealed negative results for 19 out of the 30 patients with haematologic malignancies who were ELISA positive. Nevertheless, there was no significant correlation between the PCR positivity rate and the specific type of cancer^32^. It was plausible that several limitations could have influenced the results obtained, the first was the limited sample size and the financial constraints. The second was that we did not use the avidity test or *T. gondii* IgA. The authors did not use the "area of residence" as a matching criteria between the groups. General limitations of the molecular work like shortages of reagents due to high cost are considered. Our study was limited by being a single centre institutional study including patients attending Sohag University Hospitals which could be the reason that there were relatively few *Toxoplasma* DNA detection rates. Very few reports have been published concerning the sero-molecular epidemiology and genotypic characterization of *Toxoplasma* strains in cancer patients in Egypt.

## Conclusions

*T. gondii* is highly prevalent in cancer patients undergoing chemotherapy and the healthy controls as well in Sohag, Upper Egypt. This work provides a baseline information on *T. gondii* sero-molecular epidemiology. Further large-scale research is indispensable. PCR is strongly recommended to complement serology in the diagnosis of acute or reactivated *T. gondii* infection in cancer patients. B1 nested PCR was found to be equivalent to RE PCR. Nevertheless, further thorough research must be implemented.

## Electronic supplementary material

Below is the link to the electronic supplementary material.

Supplementary Material 1.

## Data Availability

All data supporting the findings of this study are available within the paper and its Supplementary Information.

## References

[1] Q Liu ZD Wang SY Huang XQ Zhu 2015 Diagnosis of toxoplasmosis and typing of *Toxoplasma gondii* Parasit. Vectors 8 292 10.1186/s13071-015-0902-6 26017718 10.1186/s13071-015-0902-6 PMC4451882

[2] C Yoon YS Ham WJ Gil CS Yang 2024 Exploring the potential of Toxoplasma gondii in drug development and as a delivery system Exp. Mol. Med. 56 2 289 300 10.1038/s12276-024-01165-7 38297164 10.1038/s12276-024-01165-7 PMC10907749

[3] ZD Wang HH Liu ZX Ma HY Ma ZY Li ZB Yang XQ Zhu B Xu F Wei Q Liu 2017 *Toxoplasma gondii* infection in immunocompromised patients: A systematic review and meta-analysis Front. Microbiol 8 389 10.3389/fmicb.2017.00389 28337191 10.3389/fmicb.2017.00389 PMC5343064

[4] D Andreou 2024 Toxoplasma gondii infection associated with inflammasome activation and neuronal injury Sci. Rep. 14 1 5327 10.1038/s41598-024-55887-9 38438515 10.1038/s41598-024-55887-9 PMC10912117

[5] C Dard H Fricker-Hidalgo MP Brenier-Pinchart H Pelloux 2016 Relevance of and new developments in serology for toxoplasmosis Trends Parasitol. 32 6 492 506 10.1016/j.pt.2016.04.001 27167666 10.1016/j.pt.2016.04.001

[6] S Scerra H Coignard-Biehler F Lanternier 2013 Disseminated toxoplasmosis in non-allografted patients with hematologic malignancies: report of two cases and literature review Eur. J. Clin. Microbiol. Infect. Dis. 32 1259 1268 10.1007/s10096-013-1879-8 23595587 10.1007/s10096-013-1879-8

[7] J Flegr 2015 Neurological and neuropsychiatric consequences of chronic *Toxoplasma* infection Curr. Clin. Micro. Rep. 2 163 172 10.1007/s40588-015-0024-0

[8] MK Hegazy SI Awad NE Saleh MM Hegazy 2020 Loop mediated isothermal amplification (LAMP) of toxoplasma DNA from dried blood spots Exp. Parasitol. 211 2 10786910.1016/j.exppara.2020.107869 32119931 10.1016/j.exppara.2020.107869

[9] RHD Ybañez AP Ybañez Y Nishikawa 2020 Review on the current trends of toxoplasmosis serodiagnosis in humans Front. Cell Infect. Microbiol. 10 5 1 18 10.3389/fcimb.2020.00204 32457848 10.3389/fcimb.2020.00204 PMC7227408

[10] GSG Zeedan 2022 Rapid diagnosis of *Toxoplasma gondii* using loop-mediated isothermal amplification assay in camels and small ruminants Beni-Suef Univ. J. Basic Appl. Sci. 11 1 10.1186/s43088-021-00184-x

[11] A Rostami P Karanis S Fallahi 2018 Advances in serological, imaging techniques and molecular diagnosis of toxoplasma gondii infection Infect 46 3 303 315 10.1007/s15010017-1111-3 10.1007/s15010-017-1111-3 29330674

[12] P Mousavi 2018 Detection of Toxoplasma gondi in acute and chronic phases of infection in immunocompromised patients and pregnant women with real-time PCR assay using TaqMan fluorescent probe Iran. J. Parasitol. 13 3 373 381 30483328 PMC6243173

[13] F Robert-Gangneux S Belaz 2016 Molecular diagnosis of toxoplasmosis in immunocompromised patients Curr. Opin. Infect. Dis. 29 4 330 339 10.1097/QCO.0000000000000275 27191201 10.1097/QCO.0000000000000275

[14] J Bajnok 2019 High frequency of infection of lung cancer patients with the parasite *Toxoplasma gondii* ERJ. Open Res. 5 2 00143 02018 10.1183/23120541.00143-2018 31149623 10.1183/23120541.00143-2018 PMC6536861

[15] JL Burg CM Grover P Pouletty JC Boothroyd 1989 Direct and sensitive detection of a pathogenic protozoan *Toxoplasma gondii*, by polymerase chain reaction J. Clin. Microbiol. 27 8 1787 1792 2768467 10.1128/jcm.27.8.1787-1792.1989 PMC267672

[16] WL Homan M Vercammen J Braekeleer De H Verschueren 2000 Identification of a 200- to 300-fold repetitive 529 bp DNA fragment in *Toxoplasma gondii*, and its use for diagnostic and quantitative PCR Int. J. Parasitol. 30 1 69 75 10.1016/s0020-7519(99)00170-8 10675747 10.1016/s0020-7519(99)00170-8

[17] JR Landis GG Koch 1977 The measurement of observer agreement for categorical data Biometrics 1 159 174 10.2307/2529310 843571

[18] SA Hosseini 2021 Toxoplasmosis among cancer patients undergoing chemotherapy: A population study based on the serological, molecular, and epidemiological aspects Trans. R. Soc. Trop. Med. Hyg. 115 6 677 686 10.1093/trstmh/traa112 33130887 10.1093/trstmh/traa112

[19] N Kalantari 2018 Association between Toxoplasma gondii exposure and paediatrics haematological malignancies: A case-control study Epidemiol. Infect. 146 15 1896 1902 10.1017/S0950268818002194 30092850 10.1017/S0950268818002194 PMC6452987

[20] M Rouatbi 2019 *Toxoplasma gondii* infection and toxoplasmosis in North Africa: A review. Infection par *Toxoplasma gondii* et toxoplasmose en Afrique du Nord: synthèse Parasite 10.1051/parasite/2019006 30767889 10.1051/parasite/2019006 PMC6376878

[21] NM Abdel-Aziz AA Hassanien MI Arafa 2020 Detection of Toxoplasma gondii in aborted women and meat of slaughtered sheep and cattle in Sohag city, upper Egypt Adv. Anim. Vet. Sci. 8 6 680 686 10.17582/journal.aavs/2020/8.6.680.686

[22] S Hussien M Mousa MMA El-mawgod A Sobhy 2017 Seroprevalence of Toxoplasma gondii among primigravida women J. Egypt. Soc. Parasitol. 47 2 381 388 10.21608/jesp.2017.77791

[23] H Fricker-Hidalgo 2013 Toxoplasma seroconversion with negative or transient immunoglobulin M in pregnant women: myth or reality? A French multicenter retrospective study J. Clin. Microbiol. 51 7 2103 2111 10.1128/JCM.00169-13 23616461 10.1128/JCM.00169-13 PMC3697685

[24] O Villard 2016 Serological diagnosis of *Toxoplasma gondii* infection recommendations from the French national reference center for toxoplasmosis Diagn. Microbiol. Infect. Dis. 84 22 33 10.1016/j.diagmicrobio.2015.09.009 26458281 10.1016/j.diagmicrobio.2015.09.009

[25] F Peyron M Wallon F Kieffer G Graweg 2016 Toxoplasmosis JS Remington JO Klein CB Wilson V Nizet YA Maldonado Eds Infectious Diseases of the Fetus and Newborn Infant Elsevier Saunders Philadelphia, USA 949 1042

[26] A Imam 2017 Serologic evidence of *Toxoplasma gondii* infection among cancer patients. A prospective study from Qassim region Saudi Arabia Saudi Med. J. 38 3 319 321 10.15537/smj.2017.3.18535 28251231 10.15537/smj.2017.3.18535 PMC5387912

[27] NES Mostafa 2018 The relationship between toxoplasmosis and different types of human tumors J. Infect. Dev. Ctries. 12 2 137 141 10.3855/jidc.9672 31825916 10.3855/jidc.9672

[28] R Abdel Malek 2018 Toxoplasmosis an overlooked disease: Seroprevalence in cancer patients Asian Pac. J. Cancer Prev. 19 7 1987 1991 10.22034/APJCP.2018.19.7.1987 30051689 10.22034/APJCP.2018.19.7.1987 PMC6165665

[29] A Saad 2020 Role of IgG avidity test in diagnosis of Toxoplasmosis among immunocompromised patients Egypt J. Med. Microbiol. 29 2 25 31 10.21608/ejmm.2020.250012

[30] AA Elblihy SM Alhusseiny SA Elmabood SN El-beshbishi 2023 Toxoplasma gondii seropositivity among Egyptian children with haematological malignancies Parasitol. United J. 16 1 57 63 10.21608/puj.2023.199269.1204

[31] MI Ali WM Abd El Wahab DA Hamdy A Hassan 2019 Toxoplasma gondii in cancer patients receiving chemotherapy: Seroprevalence and interferon gamma level J. Parasit. Dis. 43 3 464 471 10.1007/s12639019-01111-9 31406412 10.1007/s12639-019-01111-9 PMC6667530

[32] TM Shawa HA El-taweel MA El-gammal SS Khalil S Heba 2023 Toxoplasmosis in adult patients with haematologic malignancy: Seroprevalence of anti- Toxoplasma antibodies and molecular diagnosis Parasitol. United J. 16 1 79 86 10.21608/puj.2023.203903.1209

[33] HM Khalil 1991 Opportunistic parasitic infections in immunocompromised hosts J. Egypt Soc. Parasitol. 21 3 657 668 1765676

[34] M Alim S Ozcelik N Ozpinar 2018 Seroprevalence of anti-Toxoplasma gondii antibodies in patients receiving cancer treatment Cumhuriyet Med. J. 40 1 19 24 10.7197/223.vi.387046

[35] IM Al-Tameemi B Hashim Abdullah S Jabbar Raisan 2019 Seroprevalence of *Toxoplasma gondii* among cancer patients in Basrah province/Iraq World J. Pharm. Res. 8 1 193 199 10.20959/wjpr20191-13808

[36] W Cong 2015 Toxoplasma gondii infection in cancer patients: Prevalence, risk factors, genotypes and association with clinical diagnosis Cancer Lett. 359 2 307 313 10.1016/j.canlet.2015.01.036 25641340 10.1016/j.canlet.2015.01.036

[37] NS Hijjawi 2018 Seroprevalence of *Toxoplasma gondii* in cancer patients admitted to hospitals of the royal medical services in Jordan Jordan J. Biol. Sci. 11 5 537 540

[38] DF Ahmed EJ Saheb 2017 The association of *Toxoplasma gondii* infection in breast and colorectal cancer patients Int. J. Clin. Oncol. 2 4 86 92 10.11648/j.ijcocr.20170204.13

[39] A Abdollahi 2022 Toxoplasma gondii infection / exposure and the risk of brain tumors: A systematic review and meta-analysis Cancer Epidemiol. 77 10211910.1016/j.canep.2022.102119 35152168 10.1016/j.canep.2022.102119

[40] Y Huang 2016 Is *Toxoplasma gondii* infection a risk factor for leukemia? An evidence-based meta-analysis Med. Sci. Monit. 22 1547 1552 10.12659/msm.897155 27155015 10.12659/MSM.897155 PMC4917333

[41] L Wang 2015 Seroprevalence and genetic characterization of *Toxoplasma gondii* in cancer patients in Anhui Province, Eastern China Parasit. Vectors 10.1186/s13071-015-0778-5 25889184 10.1186/s13071-015-0778-5 PMC4379604

[42] N Zhou 2019 Seroprevalence and risk factors of *Toxoplasma gondii* infection in children with leukemia in Shandong Province, Eastern China: A case-control prospective study PeerJ 7 e6604 10.7717/peerj.6604 30886781 10.7717/peerj.6604 PMC6420808

[43] JA Vargas-Villavicencio I Cañedo-Solares D Correa 2022 Anti *Toxoplasma gondii* IgM long persistence: What are the underlying mechanisms? Microorganisms 10.3390/microorganisms10081659 36014077 10.3390/microorganisms10081659 PMC9415799

[44] R Dhakal 2015 Significance of a positive toxoplasma immunoglobulin M test result in the United States J. Clin. Microbiol. 53 11 3601 3605 10.1128/JCM.01663-15 26354818 10.1128/JCM.01663-15 PMC4609698

[45] MN Khabaz L Elkhateeb J Al-Alami 2011 Reactivation of latent *Toxoplasma gondii* in immunocompromised cancer patients Comp. Clin. Path. 20 2 183 186 10.1007/s00580-010-0975-8

[46] A Gashout 2016 Molecular diagnosis of *Toxoplasma gondii* infection in Libya BMC Infect. Dis. 16 157 10.1186/s12879016-1491-5 27083153 10.1186/s12879-016-1491-5 PMC4833959

[47] F Robert-Gangneux 2015 Molecular diagnosis of toxoplasmosis in immunocompromised patients: A 3-year multicenter retrospective study J. Clin. Microbiol. 53 5 1677 1684 10.1128/JCM.03282-14 25762774 10.1128/JCM.03282-14 PMC4400795

[48] C Gomez 2019 Dual-target, real-time PCR for the diagnosis of intraocular *Toxoplasma gondii* infections Br. J. Ophthalmol. 103 4 569 572 10.1136/bjoph.lmol-2018-313064 30636207 10.1136/bjophthalmol-2018-313064 PMC6691874

[49] K Sardarian 2019 Detection of *Toxoplasma gondii* B1 gene in placenta does not prove congenital toxoplasmosis Hum. Antibodies 27 1 31 35 10.3233/HAB-180346 30103311 10.3233/HAB-180346

[50] HK Valian 2020 Comparison of the RE-529 sequence and B1 gene for Toxoplasma gondii detection in blood samples of the at-risk seropositive cases using uracil DNA glycosylase supplemented loop-mediated isothermal amplification (UDG-LAMP) assay Microb. Pathog. 140 10393810.1016/j.micpath.2019.103938 31862390 10.1016/j.micpath.2019.103938

[51] JM Costa 2013 Direct genotyping of Toxoplasma gondii from amniotic fluids based on B1 gene polymorphism using minisequencing analysis BMC Infect. Dis. 13 552 10.1186/1471-2334-13-552 24252188 10.1186/1471-2334-13-552 PMC4225555

[52] S Fallahi 2014 Comparison of the RE and B1 gene for detection of Toxoplasma gondii infection in children with cancer Parasitol. Int. 63 1 37 41 10.1016/j.parint.2013.08.005 23993996 10.1016/j.parint.2013.08.005

[53] F Veronesi 2017 Comparison of PCR assays targeting the multi-copy targets B1 gene and 529 bp repetitive element for detection of Toxoplasma gondii in swine muscle Food Microbiol. 63 213 216 10.1016/j.fm.2016.11.022 28040171 10.1016/j.fm.2016.11.022

[54] HEM Eldeek 2017 Toxoplasma genotyping in congenital toxoplasmosis in Upper Egypt: Evidence of type I strain Parasitol. Res. 116 9 2393 2406 10.1007/s00436-017-5541-8 28668985 10.1007/s00436-017-5541-8

[55] EF Botein 2019 Serological and molecular screening of umbilical cord blood for *Toxoplasma gondii* infection Transpl. Infect. Dis. 21 4 e1311710.1111/tid.13117 31102567 10.1111/tid.13117

[56] MP Brenier-Pinchart 2015 Molecular diagnosis of toxoplasmosis: Value of the buffy coat for the detection of circulating *Toxoplasma gondii* Diagn. Microbiol. Infect. Dis. 82 4 289 291 10.1016/j.diagmicrobio.2015.04.004 25957458 10.1016/j.diagmicrobio.2015.04.004

[57] R Gutierrez-Loli 2019 Development of a novel protocol based on blood clot to improve the sensitivity of qPCR detection of toxoplasma gondii in peripheral blood specimens Am. J. Trop. Med. Hyg. 100 1 83 89 10.4269/ajtmh.17-0920 30457102 10.4269/ajtmh.17-0920 PMC6335924

[58] Edvinsson, B., Lappalainen, M., Evengård, B., ESCMID Study Group for Toxoplasmosis 2006 Real-time PCR targeting a 529-bp repeat element for diagnosis of toxoplasmosis Clin. Microbiol. Infect. 12 2 131 136 10.1111/j.1469-0691.2005.01332.x 16441450 10.1111/j.1469-0691.2005.01332.x

[59] T Wahab B Edvinsson D Palm J Lindh 2010 Comparison of the AF146527 and B1 repeated elements, two real-time PCR targets used for detection of *Toxoplasma gondii* J. Clin. Microbiol. 48 2 591 592 10.1128/JCM.01113-09 19940050 10.1128/JCM.01113-09 PMC2815584

[60] C Pomares 2020 Is real-time PCR targeting rep 529 suitable for diagnosis of toxoplasmosis in patients infected with non-type II strains in North America? J. Clin. Microbiol. 58 2 e01223 e1319 10.1128/JCM.01223-19 31694976 10.1128/JCM.01223-19 PMC6989067

[61] HA Al-toban 2019 Molecular detection of Toxoplasma gondii in a sample of Iraqi patients with acute leukemia and stem cell transplantation Iraqi J. Hematol. 8 38 44 10.4103/ijh.ijh

[62] S Abolghasemi 2022 The prevalence of early toxoplasmosis after bone marrow transplantation using PCR: A study in Iranian patients Infect. Disord. Drug Targets 22 7 62 66 10.2174/1871526522666220622102543 35747960 10.2174/1871526522666220622102543

[63] SM Elzeky N Nabih AA Abdel-Magied DS Abdelmagid AE Handoussa MM Hamouda 2022 Seroprevalence and genetic characterization of *Toxoplasma gondii* among children with neurodevelopmental disorders in Egypt J. Trop. Med. 2022 2343679 10.1155/2022/2343679 35669051 10.1155/2022/2343679 PMC9166983

[64] N Arefkhah 2019 Seroprevalence and risk factors of *Toxoplasma gondii* infection among cancer and hemodialysis patients in southwest Iran Clin. Epidemiol. Glob. Health. 7 4 596 599 10.1016/j.cegh.2019.01.007

[65] S Boughattas 2010 Direct genotypic characterization of *Toxoplasma gondii* strains associated with congenital toxoplasmosis in Tunisia (North Africa) Am. J. Trop. Med. Hyg. 82 6 1041 1046 10.4269/ajtmh.2010.09-0691 20519598 10.4269/ajtmh.2010.09-0691 PMC2877409

[66] Ivovic, V., Vujanic, M., Zivkovic, T., Klun, I., Djurkovic-Djakovic, O. Molecular detection and genotyping of Toxoplasma gondii from clinical samples. In: *Toxoplasmosis - Recent Advances*, (Eds Djurković Djaković. O.) 103–120 (InTech, Rijek, Croatia, 2012). 10.5772/50830.

[67] F Robert-Gangneux ML Dardé 2012 Epidemiology of and diagnostic strategies for toxoplasmosis Clin. Microbiol. Rev. 25 2 264 296 10.1128/CMR.05013-11 22491772 10.1128/CMR.05013-11 PMC3346298

[68] F Nakashima 2020 Serum IgG anti-*Toxoplasma gondii* antibody concentrations do not correlate nested PCR results in blood donors Front. Cell Infect. Microbiol. 9 461 10.3389/fcimb.2019.00461 31993377 10.3389/fcimb.2019.00461 PMC6970978

[69] O El-Geddawi M El-Sayad N Sadek N Hussien M Ahmed 2016 Detection of *T. gondii* infection in blood donors in Alexandria, Egypt, using serological and molecular strategies Parasitol. United J. 9 1 24 10.4103/1687-7942.192992

