# Supplementary material for: Serological and molecular detection of Toxoplasma Gondii among cancer patients in Sohag, Upper Egypt: a case-control study
Source: Sci Rep. 2025 Feb 12;15:5236. doi: 10.1038/s41598-025-88680-3 (PMC11822034; doi:10.1038/s41598-025-88680-3)
Supplement: Supplementary file 1 — Supplementary Material 1. [file 41598_2025_88680_MOESM1_ESM.pdf]

Supplementary data

Raw gel images

S1

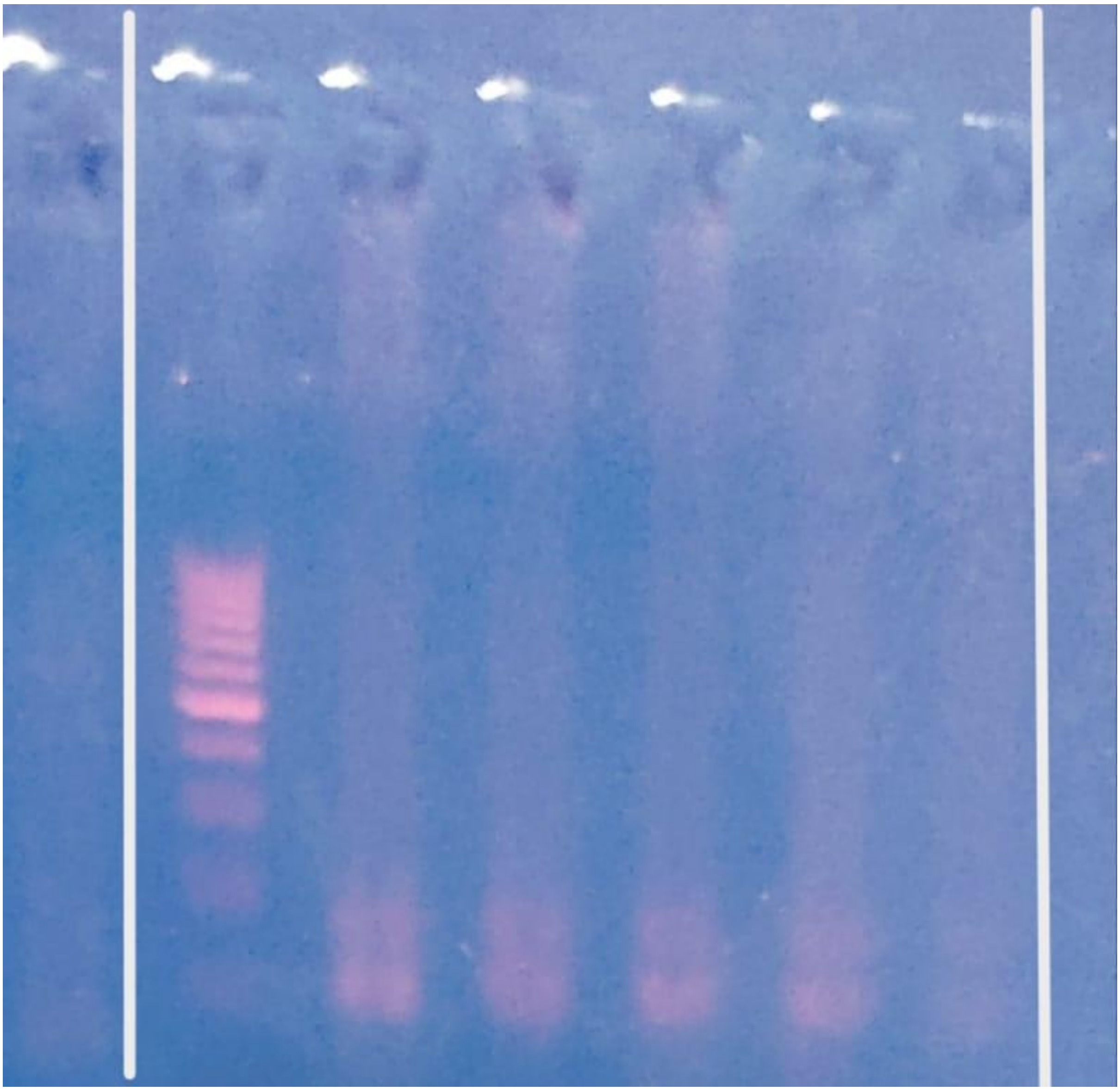

Toxoplasma B1 gene

The white lines indicate the crop sites (Fig 3 in the manuscript).

S2

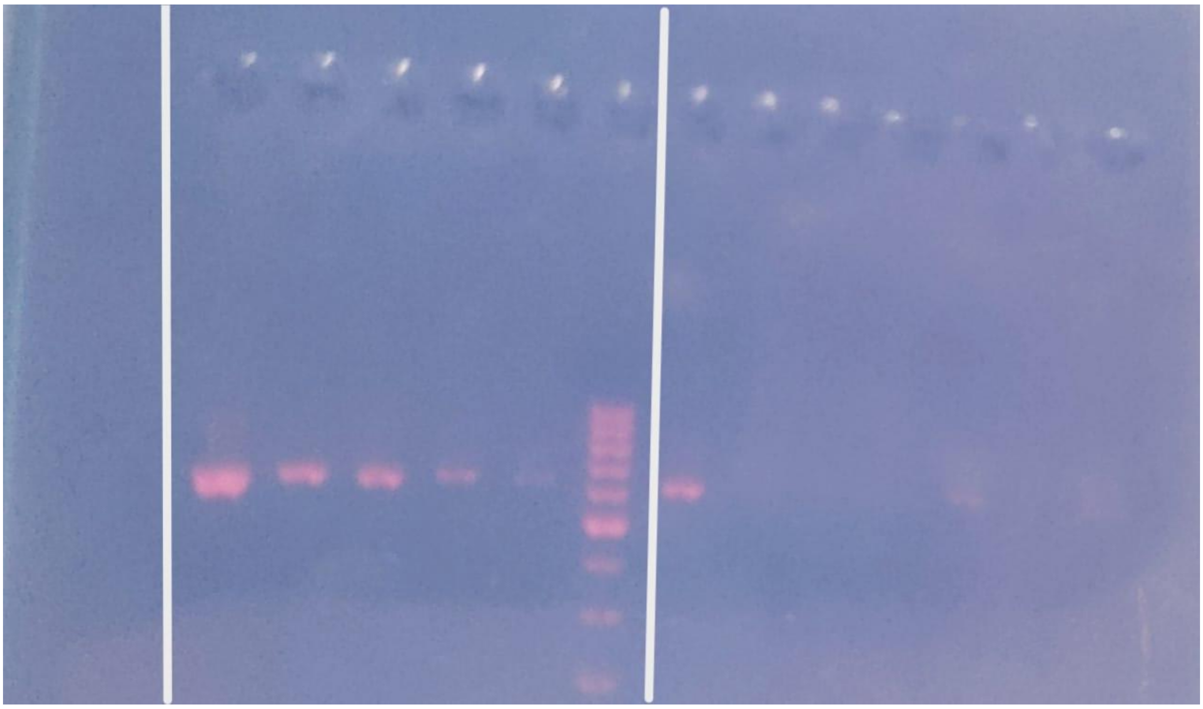

Toxoplasma RE gene

The white lines indicate the crop sites (Fig 4 in the manuscript).
